# Lectures on Inflammation and Ulceration of the Cervix Uteri—In Answer to the Croonian Lectures of Dr. Charles West, on the Same Subject

**Published:** 1855-03

**Authors:** Henry Miller

**Affiliations:** Professor of Obstetric Medicine in the Medical Department of the University of Louisville


### MEDICINE AND SURGERY.

MARCH, 1855. NEW SERIES. VOL. III.—NO. 3.

### ART. I.—LECTURES ON INFLAMMATION AND ULCERATION OF THE CERVIX UTERI—IN ANSWER TO THE CROONIAN LECTURES OF DR. CHARLES WEST, ON THE SAME SUBJECT.

BY HENRY MILLER, M. D., PROFESSOR OF OBSTETRIC MEDICINE IN THE MEDICAL DEPARTMENT OF THE UNIVERSITY OF LOUISVILLE.

### LECTURE III.

IN the two preceding lectures, it has, I trust, been satisfactorily established that the neck is no unimportant portion of the womb; and that it has special functions to perform as well as the body, equally indispensable to the grand function which is committed to them jointly. Any attempt to degrade the one and exalt the other is, therefore, a perversion of sound physiology, and it is manifest that the tendency of Dr. WEST's investigations is toward the limited and contracted views, which he reprobates in others, for while they may probably attach too much importance to the neck, he has himself certainly over-estimated the physiological and pathological rank of the body. To such an excess is he led away by this ultraism, that he does not, as we have seen, hesitate to speak of the body, exclusive of the neck, as the *uterus*, and to designate discharges proceeding from it *uterine*, as though discharges from the neck ought to be distinguished by some other appellation.

It has furthermore been shown that the neck may be involved in inflammation without any participation on the part of the body, which is defended, in a good degree, from the encroachment of the morbid action by the shutting of the portal of communication between them,—the *cervico-uterine* or *internal orifice.* No reason can be assigned, and certainly none has been by Dr. WEST, why such imflammation may not be an original or primary affection, independent of inflammation or other morbid action of the body, and it is as little questionable that the neck being inflamed, in this isolated manner, may give rise to uterine symptoms as readily as may the body—their sympathies and morbid reactions being identical.

Having annihilated the cervix, Dr. WEST was forced to cast about and find causes of uterine maladies which expend their disturbing influence, by a sort of elective affinity, upon the body of the organ, and likewise to seek an explanation of the success of local remedies applied to the cervix in relieving and permanently curing many cases of uterine disease. This is the scope of his third and concluding lecture, to which I shall, to day, direct your attention. In the commencement of it, Dr. WEST manifests a just appreciation of the difficulties before him. The cervical doctrine having been abolished by him, he was pained to announce to his auditors that he had no theory to offer as a substitute that would afford so simple and apparently so felicitous an explanation of the complex morbid processes and their cure, as it had appeared to do. O, no, he can put forward no such pretensions, for, in his deep musings on the arcana of nature, he had, *mirabile dictu*, discovered that the different symptoms ascribed to ulceration of the os uteri may arise in reality from very various causes,—"at one time they attend on constitutional disorder, at another on *some ailment* of the sexual system, and that ailment by no means the same in every instance." So, then, it seems that the sexual organs actually live under a constitution and they are obnoxious to diversified "ailments." An illustration of their subjection to the constitution is fetched from the case of the chlorotic girl, and I have no particular fault to find with it, except that I have not known such abundant leucorrheal discharges to attend, as are mentioned by Dr. WEST. In the disordered digestion, malassimilation, and defective hematosis, that characterize chlorosis, there is adequate cause for the imperfect performance or the non-establishment of the menstrual function, and the proper remedies for the sexual atony are to be sought in all such measures, hygienic and therapeutical, as improve the general health. This, it may be presumed, would be conceded even by Dr. Bennet.

Dr. West draws other illustrations of constitutional agency from the influence over the sexual functions of hepatic and nephritic disease, and from the gouty and rheumatic diatheses,—all of which, as he shows, may variously derange menstruation and give rise to leucorrheal discharges. Nor do I doubt that, in such instances, the appropriate remedies may be colchicum, iodide of potash, diaphoretics and sedatives, alkalies and vegetable bitters, so highly lauded by him, without any allusion to topical treatment. Dr. West has, however, jumbled together things that are incongruous, which ought to be kept separate, when viewed as remote causes of sexual maladies. Functional and organic diseases of other organs extend their morbid influence to the uterine system in a quite different manner from diseases or diatheses, which involve the whole constitution, such as gout, rheumatism, anæmia, plethora, scrofula, syphilis, &c. In the former case, the perturbing influence is propagated through the channel of sympathy, growing out of nervous and vascular inter-communication; in the latter, the constitutional vice, whatever it may be, reaches the sexual, in common with all other organs. In reference to the former, it cannot be doubted that they may be produced by the reciprocal morbid influence of functional and organic diseases of the sexual organs, and hence there are few cases of such disease, of long standing, that do not sympathetically affect the digestive and urinary organs. The problem of disease thus becomes a complex one, and it may not always be easy to find the first link in the morbid chain; nevertheless, when it is ascertained beyond all doubt that the sexual organs are the seat of chronic inflammation and its results, it may safely be inferred that, as a general rule, whatever functional derangemeuts of other organs may attend are purely sympathetic.

Again: when the uterus is seriously involved by constitutional diatheses or diseases, it will generally be found that it has become the seat of correspondent positive lesion, requiring local, in conjunction with constitutional, treatment. Such is assuredly the fact in regard to scrofula and syphilis, the womb being liable in common with other parts to the specific inflammation which these maladies set up in their progress. In such cases, local remedies are as much indicated as in scrofulous or syphilitic inflammation of the eyes or fauces. The pathological importance of ulceration of the os uteri may be diminished when it is only one of many local affections that owe their paternity to a constitutional cause, but even then it is not unimportant nor can the speculum be dispensed with in its treatment. Could Dr. WEST, therefore, succeed in demonstrating that cervicitis, or as he prefers to denominate it, ulceration of the os uteri, is never any thing but a secondary affection, he would not despoil the speculum of its trophies, for whatever may be its origin, topical applications may be required for its subjugation.

Having assigned to the "constitution" its share of the spoils wrested from the *neck*, Dr. WEST proceeds to bestow the remnant upon his favorite *body* of the womb,—leaving the neck completely shorn of the honors with which it had been bedecked by such fantastic pathologists as BENNET, WHITEHEAD and a mob of French authors. In a large proportion of cases, uterine ailments are attributable to marriage, pregnancy, abortion or delivery,—Dr. WEST says in 40 to 50 per cent., and a table was brought forward in his second lecture to prove this allegation, which I did not think it necessary to notice. These are indubitably fruitful sources of uterine disease, acknowledged as such by pathologists on both sides of the question under discussion. As to the special lesion produced by them, Dr. WEST alleges that "according to the opinion of some observers," these causes act by producing inflammation of the cervix uteri and consequent ulceration of its orifice, but he thinks a different explanation is not only allowable but requisite. What his explanation is you can be at no loss to anticipate; it is, of course, such an one as will give to the body preeminence over the neck and crown it with pathological laurels; body *versus* neck. To illustrate and enforce his views, Dr. WEST selects the case of a puerperal woman, who leaves her bed too soon or makes some injurious exertion, while the uterus is still heavy, its vessels large, and the process of involution of its tissue is still incomplete. The immediate consequence of her indiscretion is persistence of the lochial discharge, converted into uterine hemorrhage at the next menstrual period, and this succeeded by abundant secretion from the mucous membrane. The hemorrhage recurs periodically, with wasting leucorrhea in the intervals, by which the blood is impoverished and the health is enfeebled. In such a case, we shall probably find some prolapsus and increased size of the uterus, its orifice open and perhaps somewhat abraded. Whence now in this case, asks Dr. West, comes the hemorrhage? From the whole extent of the genital mucous membrane it may be, and doubtless that portion of it lining the body furnishes the greater part of it. And I do not dissent from his opinion that the general uterine congestion is the essential malady, at least in the onset, which may be most successfully combated by the remedies prescribed by him, namely, rest, tonics, and the cold douche, to which may be added ergot in moderate doses, repeated several times in the day, to promote contraction of the uterine fibres. Inflammation may supervene upon such a state, marked by extreme tenderness of the womb, acute pain, purulent discharges, often tinged with blood, and constitutional derangement, leaving, on its subsidence, the uterine tissue harder in texture than it was previously. Dr. West points out the liability of such uterine inflammation to relapse; yielding apparently to remedies for the time being, but recurring over and over again in spite of treatment.

Even after the advent of inflammation, he sees nothing in the condition of the neck worthy of special notice; its lining membrane doubtless participates in the general congestion and its follicles *probably* secrete more actively, but no one can suppose that a slight abrasion around its orifice, should it chance to be present, is here a matter of any consequence. Dr. Bennet makes a very different report of the pathology of such cases. In an article on menorrhagia in the *London Lancet** speaking of menorrhagia after parturition, he says: "The continued and obstinate hemorrhage which is often observed after parturition, both before and after the return of menstruation, is nearly always complicated with

* Vol. I. page 381, 1852.

and occasioned by inflammatory ulceration of the neck of the uterus, with or without disease of the body of the uterus." He allows that the blood escapes from the lining membrane of the uterine cavity, as well as from the ulcerated surface; he has frequently seen the blood oozing from the latter and often checked it instantaneously by freely cauterizing with the solid nitrate of silver, the *entire* ulcerated surface, both internally and externally to the os uteri. My own observations accord, in the main, with those of Dr. BENNET, who, it will be perceived, does not exclude the body from all participation but asserts the special prominence of cervical disease, of which, as a general rule, there can be no doubt. Dr WEST himself unwittingly corroborates this fact, in the narrative he gives of one of his cases, that of a married woman, aged 41, admitted several times into St. Bartholomew's Hospital, who had suffered two abortions and given birth to one child at the full period, after a most lingering labor. Her symptoms, which were those of most uterine maladies, dated from the time of her tedious labor. At her first admission into the Hospital, "the uterus was found to be rather low down, but not much enlarged, though very tender; the cervix uteri was indurated, somewhat elongated, and very painful; and the os uteri, which was small and circular, presented no trace of abrasion, either affecting its lips or extending into the canal of the cervix, though the congestion of that part was very marked." She was, for the time, relieved by rest, frequent local leeching, and sedatives. Relapsing in less than twelve months, she returned to the Hospital and underwent the same treatment, and we are emphatically told that "the os uteri on this occasion also presented no abrasion, though frequent examinations were made with the speculum to ascertain the fact." Again she relapsed and returned to the Hospital, and "on her admission, there was the same intense congestion of the os uteri as on former occasions, with a very abundant, highly offensive, purulent discharge, slightly tinged with blood from its interior; the womb itself being low down, somewhat larger than natural, and the cervix large, hard, swollen, and intensely tender; but no trace of abrasion of the os was perceptible."

In his comments on this case, Dr. WEST draws special attention to the fact that, although the symptoms were exceedingly severe and recurred again and again, for the space of three years, there was no ulceration of the os uteri or of the canal of the cervix, and he sagaciously conjectures that there must have been "some inflammatory affection of the mucous membrane of her uterus," by which is meant, of course, the *corpus*,—for, hang the neck, it has nothing to do with the uterus. It would be difficult, I submit, to find, in the whole compass of the recorded experience of the profession, a case looked at through a more refracting medium; it was manifestly one of cervical inflammation, and it is obvious that Dr. West must imagine either that all this controversy is about abrasion of the os uteri, or that there can be no inflammation where there is no abrasion. Mark the frequent examinations with the speculum, for no other assigned purpose than to ascertain the fact and make sure of it that there was no abrasion! It is true, the cervix was "*large, hard, swollen, and intensely tender*," from which an ordinary observer might have inferred that it was inflamed, but no, the philosophical Croonian might say, there was, I will be sworn, no trace of "abrasion" of the os.

Other cases might be adduced by him, which, he thinks, admit of the same interpretation, namely, that the interior of the uterus suffers first and chiefly, and whatever may befall the neck is but incidental and unessential. It is from this grand uterine interior, according to him, that the greater part of the leucorrheal, and all the hemorrhagic discharge is furnished; in short, it is the great seat and centre of the most important pathological as well as physiological processes. It is easy to dogmatize after this fashion, but then unfortunately we have his own admission, towards the conclusion of his third lecture, that it is only *probable* that such is the source of leucorrheal discharges, as we have not, in his opinion, the means of ascertaining with positive certainty its real origin! From this opinion I beg leave to dissent; we can, I think trace this stream to its fountain and it is susceptible of proof that, in a large majority of cases, the fountain breaks forth in the neck and not in the body of the womb. Owning such imperfection of diagnosis, how could Dr. West pretend to settle so definitely and authoritatively, as he did by table in his second lecture, the source of leucorrheal discharges? Then it was asserted that the source of the discharge had been ascertained in the cases tabulated and it was traced to the uterine body, the neck contributing little or none.

Although he has only *probability* in favor of his own opinion, concerning the origin of leucorrhea, Dr. WEST, curiously enough, declares that the weight of proof seems to him "to lie upon those who see in the leucorrhea only a hyper-secretion from the glandular apparatus of the cervix uteri." Under protest of his right to demand it, I accept the *onus probandi* and shall attempt to show that leucorrheal discharges, in most cases of chronic uterine affections, proceed from the cervix and are the product of inflammatory or ulcerative disease. It is needless to say that no pathologist holds the doctrine, imputed to a whole class by Dr. WEST, that leucorrhea is only a *hyper-secretion* from the glandular apparatus of the cervix.

In favor of the cervical origin of leucorrheal discharges, in most cases of uterine profluvia, it may be urged—

1. *That such is their source, according to the observations of* Mr. WHITEHEAD, *already referred to, in the leucorrhea of pregnancy.* Independently of these observations it might, indeed, have been inferred that the neck is the portion of the organ that furnishes them rather than the body, in consideration of the different physiological condition of the two portions during pregnancy. Whether we regard the decidua as a membrane of new formation or as the uterine mucous membrane, hypertrophied to fit it for the relations it is to bear to the ovum,—in either view, the fact is undoubted that the entire cavity of the body is occupied by the ovum and that an intimate attachment is formed between them. The whole extent of its mucous membrane is brought into union with the fœtal chorion and is made tributary to the growth of the ovum. There is then no part of it that presents a free surface, which could possibly yield a secretion, natural or morbid, that could escape by the os uteri. Not so with the cervix. It forms no connection with the ovum; its mucous surface is free and continues so even to the close of pregnancy, its follicles being naturally in a state of hyper-secretion, continually renewing the mucous plug, as fast as it dissolves in the vagina, and thus keeping the cavity of the body sealed up till its gestative work is accomplished.

In the cervical canal, therefore, there is not only a free surface, with a ready outlet, but a surface excited to exaggerated secretion, by the stimulus of pregnancy, and predisposed by this unwonted functional activity to take on morbid action. Physiology thus sanctions Mr. Whitehead's pathology of the leucorrhea of pregnancy and is irreconcilably opposed to the doctrine of Dr. West, at least in every case of the disorder occurring in the course of pregnancy. Seeing that leucorrheal discharges are very common during pregnancy, and can have no other source than the neck, it must, I think, be acknowledged that it is highly *probable* that they have the same origin when pregnancy does not exist. It being established that there is an adequate proximate source, we have no right to seek a remote one, unless it be found, by actual exploration, that the former is in a healthy condition.

2. *The character of the discharge, as seen issuing from the os uteri, may be taken as an index to its source.* According to the researches of M. Coste, * confirmed by others, the cavities of the two portions of the uterus are lined by mucous membranes differing materially in anatomical composition and structure, adapting them to the different functions they are destined to perform. This anatomical difference gives rise to a marked difference in the product of their secretory apparatus, that of the body being, according to M. Robert, † *a thin, colorless liquid, resembling badly clarified whey, which is only slightly viscid*, while that of the neck is *thick, transparent, and ropy, resembling the white of egg, and remarkably tenacious.* In relation to the mucus of the uterine body, M. Robert observes that it is not unusual, in the autopsy even of women who had not complained of uterine symptoms, to find it of a more or less red color, owing to the admixture of blood,—blood globules appearing under the microscope.

This is not surprising when it is considered that the membrane which secretes it is the periodical seat of congestion that relieves

* Histoire Générale et Particuliere du Développement des Corps Organisés.

† Archives Générales.

itself by sanguineous exudation, and that it is, beyond all other membranes of its class, prone to hemorrhage. Hemorrhagic depletion would seem to be the natural remedy of its congested state rather than hyper-secretion, which is more usual in the mucous membrane of the neck, in common with all others of the same class. Judging from the nature of the membrane and its secretion, Dr. BENNET is, I think, correct in declaring that a sero-sanguinolent discharge is as characteristic of internal metritis as the rust-colored expectoration is of pneumonia. Between such a discharge and the albuminous, tenacious mucus of the neck the difference is so great that it can hardly escape notice, and each points almost unerringly to its source.

3. *The uterine sound enables us to demonstrate the precise source of discharges from the womb.* Inflammation of either cavity of the uterus is almost invariably attended with a patulous state of the orifice by which it communicates with the vagina, the outlet of its redundant secretion. Those who have used the speculum, even in a few cases, must be familiar with this fact, as far as the external or vaginal orifice of the uterus is concerned. It is easily seen to be enlarged and quite patulous in any case of *cervicitis;* and if its canal be explored by the sound, we shall find that the *cervico-uterine* or internal orifice is either more patulous or more contracted than in a healthy state, according as the inflammation has or has not extended into the cavity of the body. If the sound pass with unusual difficulty or cannot be made to penetrate at all, we may consider such obstruction conclusive proof that the inflammation is limited to the neck; while, on the contrary, if no resistance be encountered, and the sound penetrate readily, as if there were no internal orifice but one continuous, ample channel, it is equally certain that the body is implicated.

I have satisfied myself, by such exploration aided by other signs, that inflammation of the lining membrane of the body not unfrequently exists in connection with cervicitis; but I have never met with an instance of it where the neck was unaffected. So far as my own observation goes, therefore, inflammation of the body may well be called metritis or uteritis, for it is always associated with inflammation of the neck and thus the entire organ is involved. Who will venture to affirm that inflammation of the neck always or even frequently involves the body? And yet, unless it can be established that the body is often inflamed independently of the neck, and that inflammation of the neck is readily propagated to it, Dr. West's claims in its behalf as the special and elect seat of leucorrhea cannot be allowed.

From these facts and considerations, together with his own admission, it is manifest that Dr. West has taken the liberty, not only without proof but in opposition to all that is certainly known, to assign leucorrheal discharges to the *corpus uteri.* I have no hesitation in declaring, as the result of my own experience, that *uterine leucorrhea*, in his sense of the term, is exceedingly rare, and if the cervix be excluded from all participation, I know not indeed that there is in reality any such disease at all.

After having usurped for the body of the uterus supreme importance, Dr. West endeavors to define his position, to use a political phrase, relative to the neck. "But it may not unnaturally be asked, whether I then believe that the condition of so-called ulceration of the os uteri is one of absolutely no importance, adding nothing to a patient's sufferings, in no respect protracting her illness, calling for no treatment? I do not believe this; though at the the same time disease of the os uteri is so almost invariably associated with other evident ailments of the organ as to render it very difficult to distinguish accurately one set of symptoms from the other." This is a fit introduction to the beggarly account of local affections which it pleases him to assign the cervix, and a doubt is evidently insinuated whether it is, in its own right, entitled to even these. What is meant by their being almost invariably associated with other *ailments* of the organ? If it be intended to affirm that lesion of the body, appreciable by either the touch or sight, almost invariably co-exists with them, this is contradicted by the most accurate clinical observation, according to which, as I have more than once stated, the neck is often diseased without involving the body. But if the "ailments" referred to be only some functional derangement of the body, it is readily admitted that this may and usually does occur as a consequence of cervical maladies.

Dr. West's meagre inventory of diseases of the os uteri begins with an *abraded condition*, *which*, *under some circumstances*, is capable of producing very considerable *discomfort*, and as this ought, by all means, to be known and believed, he confirms it by the *remarkable case* of a woman who was the subject of dysmenorrhea and had lived in sterile marriage with two husbands. Under the "impression" (for it could not be certainly determined in St. Bartholomew's Hospital) that the difficult menstruation might arise from a mechanical cause, a sponge tent was inserted in the os uteri and actually produced "a very distressing sense of itching referred to the uterus!" Furthermore, "on the withdrawal of the tent, the edges of the os uteri and the cervical canal, as far as it could be seen, were observed to be very red, and quite denuded of their epithelium, while a rather abundant glairy secretion was poured out from their surface." So long as the abrasion continued, and it did not disappear for *three whole days* after the removal of the tent, the sense of itching and the discharge continued, though (as the reader must feel greatly relieved to learn) with gradually diminishing severity. This, which the learned Croonian pretends to think a fair type of inflammatory affections of the cervix, resembles rather the *cacoœthes loquendi* which a little rambling talk may suffice to dissipate.

The second affection in the inventory is *a red, and coarsely granular condition of the orifice of the womb*, from which a glairy secretion is abundantly poured forth, with little or no appreciable evidence of uterine disease, for it must be remembered that the orifice is no part of the uterus. This consists, we are told, in hypertrophy of the *papillæ* and is analogous to the granular condition of the palpebral conjunctiva in purulent ophthalmia, and why, we might ask, is it a slight affair in the os uteri and a very grave one in the eyes? The third and last lesion in the list is the *granular metritis* of BOIVIN and DUGES, *i. e.* follicular inflammation of the os uteri, upon which the lecturer descants, but we need not trouble ourselves to follow him. The first of these affections, namely, the abrasion with itching, may be produced at pleasure, and will speedily subside when the irritating cause is removed; the other two are seldom met with in practice, at least with us, whilst simple inflammation, such as all organs and tissues are liable to, is exceedingly common and is much oftener observed in the neck than in the body of the womb. This is emphatically the true burden of controversy, which Dr. West would fain keep in the back-ground and from which he seeks to divert attention by putting forward pathological rarities. Cervical inflammation will not, however, always vanish at his bidding, for notwithstanding the secondary and trivial importance assigned it, we find him confessing that it does sometimes outlast the graver evils under which it arose and that it may thus cause "discomfort, leucorrhea, and slight sanguineous discharge, keeping up a perpetual disposition to uterine congestion, which but for it would subside." "That under such circumstances," he goes on to say, "a tendency to slow increase in the size of the cervix uteri should exist, is surely no matter for wonder, since the neck of the womb is more exposed to irritation of every kind than any other part of the organ; while slight though the morbid state may be, it yet is sufficient to produce some increased afflux of blood thither, whence its return is more difficult than from any other part; and we have already seen how great is the tendency in the uterine tissue under any stimulus, either natural or morbid, to some degree of that hypertrophy which, during thirty years of life, represents its highest physiological condition."

This concession looks very much like a surrender and it could scarcely be regarded in any other light, had he only granted that inflammation may arise in the neck independently of the body. But no, says Dr. West, not even a word of explanation is necessary to point out the difference between his opinions and those he has ventured to criticize, and according to which inflammation of the cervix and ulceration of the os uteri are the first and the last in uterine pathology. It seems to me that it matters little whether such a morbid state of the cervix be the first or the last, provided it be admitted that uterine derangement is kept up by it, until it is cured by topical medication.

We are thus led to the most interesting part of our discussion, viz., the treatment of uterine disease, when there is cervical inflammation or ulceration, whether it be regarded as the essential malady or an accidental complication. Dr. West enters upon this part of his subject by anticipating a question, which is rather a poser; it is this,—"how is it that such successful results have followed a course of treatment directed exclusively to the cure of the ulceration,—that the application of caustics to the os uteri has been succeeded by the restoration of the patient to health ?" Before we examine the solution offered by him, it will be well to note that he does not deny the fact of cures being wrought by those addicted to this kind of medication.

Indeed, if this treatment were all a sham, and no benefit accrued from it, the question could not be entertained for a moment. Unlike many enemies of the speculum, Dr. WEST admits that *womb-burning* is salutary or, at any rate, that it is not incompatible with the restoration of the patient to health; but like his prototypes of old, the Pharisees, who admitted the truth of miracles that could not be gainsaid but attributed them to satanic agency, he seeks an explanation of the success of the treatment he repudiates in the circumstances provided for the patient rather than in the direct caustic medication.

The collaterals, to which such remedial efficacy is awarded, are, temporary separation from the husband, rest in a recumbent position, attention to the bowels, regulation of the diet, and when convalescence ensues, a visit to the country or some watering place, if the patient's circumstances permit. The very simplicity of these salutary measures is, he alleges, a bar to their adoption, should they be recommended as in themselves the means of cure, but when enjoined only as necessary conditions of a cure by cauterization, they are willingly submitted to. As to cauterization with the nitrate of silver, the article most frequently used, the surface to which it is applied, says Dr. WEST, is covered by a thin layer of albuminous secretion, which greatly diminishes the power of the agent, so that it seldom does harm, sometimes does real good, though no more than might be attained by vaginal injections used by the patient herself!

From this condensed statement of the reply of Dr. WEST to the question, it will be perceived that he has a very exalted opinion of the efficacy of rest and of abstinence from whatever is calculated to aggravate uterine inflammation, in which I entirely concur. But are these measures curative? and will chronic inflammation of the cervix yield to hygienic treatment alone? None but a neophyte would venture to answer these questions affirmatively, and my own observation authorizes me to declare the inadequacy and consequent inefficacy of such measures; they are beneficial as adjuvants but impotent as remedies. Many patients have come under my observation who had thoroughly tested such treatment under the direction of other practitioners and had vaginal injections to boot, who nevertheless were not cured. But it will be observed that if Dr. West's appreciation of "simples" is very high, his estimate of cauterization is proportionably low, and none of his assertions or perversions, I confess, has surprised me more than his brief allusion to the nitrate of silver as a topical remedy. The efficacy of this kind of medication in kindred, I might say identical affections of other mucous membranes, is so well established that a lecturer before a learned society ought not to have called it in question, respecting uterine inflammation, unless he had proof that the genital mucous membrane is unlike all others of its class. It is true, that he does not exactly say that the nitrate is inert *per se*, but the surface of the os is shielded from its causticity by a layer of albuminous mucus,—a wise precaution, it may be supposed, of nature, foreseeing that there would be womb-burners in the last days.

Unfortunately for Dr. West's theory, however, but fortunately for the womb, this albuminous shield is rudely assaulted by every cauterizer with a formidable weapon, called by his Gallic neighbors *plumasseau de charpie*, of which he may have heard, which brushes it aside *an 'twere any* cobweb and exposes the naked surface to the contact of the nitrate crayon. There can be no doubt that the uterus may be cauterized more perfectly than the tonsils or fauces and more readily than the larynx, and certainly no reason can be imagined why the remedy should not be as efficacious when applied to it as to them. What analogy suggests experience has abundantly confirmed, for if it be true that these cases, after having resisted the best directed hygienic measures, have yielded, not once but in hundreds of instances, to cauterization, what other or stronger evidence can we obtain in favor of any remedy?

Valuable as cauterization is, however, in the treatment of inflammation of the cervix uteri, it is not the only topical remedy employed in such cases, nor is the nitrate of silver the only caustic that may be advantageously applied. The abstraction of blood directly from the affected part, by leeches or scarification, is often useful and may sometimes supersede cauterization, while the most beneficial results may often be obtained by medicinal applications that are not caustic in their operation, Still, cauterization is applicable to so many cases, where depletion is not indicated, and is so often preferable to other local medication, that we need not hesitate to admit that it is the prominent and distinctive feature of our treatment.

Whilst Dr. WEST denounces cauterization with the nitrate of silver as nugatory or simply superfluous, he thinks it would not be right to leave unnoticed other cases, in which the neck of the womb being more or less enlarged, stronger agents are employed. The stronger agent referred to is the caustic potash, which he summarily condemns on account of the pain it produces and the risk of subsequent inflammation of the uterus and its appendages. I have often used this article or its equivalent, the *potassa cum calce*, in the treatment of such cases and may, therefore, presume to know something about its immediate and remote effects. All my own observation is contradictory to the assertions of Dr. WEST, both in regard to the pain and danger of the remedy. On the first point, Dr. WEST may speak for himself, the rather, as it will plainly appear that he contradicts, in this place, what he affirmed in his first "lecture." "If the caustic be introduced, as is usually done, within the cervical canal, it is allowed that the pain produced, and which sometimes lasts for two or three days, is very intense, causing nausea or sickness, and sometimes even syncope, or occasioning extreme depression, prostrating a patient so completely as to render her unable to quit her bed or sofa for several days."

Compare this statement with what was asserted in his first lecture touching the insensibility of the neck. "The cervical canal has been forcibly dilated, it has been incised; the tissue of the cervix has been burnt with the strongest caustics, or with the actual cautery, or portions of it have been removed by the knife, generally with no injurious consequence; often with so slight a degree of constitutional disturbance, or even of local suffering, as to surprise those who advocate, little less than those who condemn, such proceedings."

But I am afraid that if Dr. WEST's consistency may be called in question, his accuracy in representing the practice of others is not unimpeachable. It is not true that the caustic potash is usually introduced into the cervical canal; far more frequently it is applied to the exterior of the neck, in cases of enlargement and induration, and there only to a small part of its superficies for the purpose of forming an issue, precisely as an issue is made upon any part of the skin. The suppurative inflammation that detaches the eschar is followed by purulent discharge, under the influence of which the morbid tissue softens and returns gradually to its healthy condition, while the body of the womb, if implicated, is ameliorated by the counter-irritation established in the neck. In those comparatively rare cases in which it may become necessary to introduce this powerful agent into the cervical canal (and I have met with such), it is never with a view to produce an eschar but to make a somewhat more powerful impression than the nitrate of silver can, or at most to cauterize very superficially. In the former case, the caustic is held in contact with the neck six or eight minutes; in the latter, not longer than as many seconds. Such at least is the procedure of Dr. Bennet, who is doubtless the butt of Dr. West's criticisms, and who, I may add, is the embodiment of the doctrine and practice he is striving to eradicate. On this point, Dr. Bennet says, "When applying potassa fusa or potassa cum calce to the cavity of the neck of the uterus, I never leave it more than a few seconds in contact with the diseased surface, as the object is not to create a slough, but profoundly to modify its vitality. I generally use the smallest cylinder, which, from its size, moves freely in the enlarged cavity, only applying it where there is evident morbid dilatation; and never beyond half or three quarters of an inch in depth, even when the disease appears to penetrate farther."

What will now be thought of the controversial fairness of Dr. West, when we find that immediately after mis-stating the surface of the cervix usually cauterized with potash, he misrepresents the intensity of it by speaking of the eschar produced by it!

I have not myself observed that the pain resulting from the application of the *potassa cum calce* is much, if any more severe than that produced by the nitrate of silver, and it has not fallen to my lot to witness a solitary instance of supervening inflammation of the uterus and its appendages. But although I might be emboldened by such experience, I do not advocate a resort to the most powerful remedy, which may, and in careless hands doubtless will do mischief, until milder and less hazardous means have been fairly tried, unless the case be such an one as the practitioner is satisfied, by experience and careful observation, will require the more powerful. The application of caustic potash to the interior of the neck is liable to be followed by contraction and even obliteration of its canal, impeding or altogether obstructing its functions, and such consequences are always to be apprehended from its injudicious management. It ought therefore, not to be practiced without all the precautions, which Dr. BENNET and other authors are at such pains to inculcate. Its liability to abuse is, however, no argument against its proper employment, and it is certainly quite indispensable in some cases. He who has not met with such, and idly fancies that the neck enjoys immunity from essential disease, reflecting only the pathological images cast upon it from the phantasmagoria of the body, has yet much to learn ere he will be qualified to combat its deep-seated lesions.

But suppose, for argument's sake, the neck and its diseases as unimportant as Dr. WEST will have them, how shall chronic inflammation of the lining membrane of the body be treated? Are constitutional remedies adequate to its subdual? or is it beyond the reach of topical medication? These are important questions, not mooted by Dr. WEST, and if, in answering them, it shall appear that this portion of the organ is accessible to topical remedies and that its diseases may be ameliorated or eradicated by them, when constitutional treatment alone is unavailable, the speculum will maintain its ground notwithstanding his covert attack upon it. From the whole tenor of his lectures we may safely infer his repugnance to the speculum, and we may confidently predict that those who share his antipathies will feel themselves wonderfully braced by the perusal of them. If other evidence of this disguised repugnance were wanting, we might point to his studied silence respecting all that has been proposed and done, in the way of local medication, with a view of curing affections of the uterine body, notoriously rebellious to every form of constitutional treatment. For aught that appears to the contrary, in his lectures, it is only necessary for disease to nestle in the *corpus uteri*, in order that it may be safe from the encroachments of the speculum and enjoy an indefinite incubation.

M. Melier is the first writer, so far as I know, who recommends pursuing inflammation to its innermost recess in the cavity of the uterus. The main design of his memoir, cited in my second lecture, was to prove that the uterus, though belonging to the class of internal organs, may be made, to all therapeutic intents and purposes, an external one, and that, by means of the improved speculum employed by him, its diseases may be treated locally with the same precision and effect as external or surgical affections. The speculum used by him was a metal conical tube with a wooden stopper, terminating at one of its extremities in a round or conical head that projects some lines at the small aperture of the tube, while the other extremity projects also in the form of a handle,—a kind of instrument novel at the time but familiar to every one at present. In the treatment of chronic inflammation of the os uteri and ulcerative disease of its exterior, M. Melier attached great importance to local baths, simple or medicated, administered through the speculum, and dressings of divers unguents spread on lint and applied to the affected part. These local baths and dressings he directs to be repeated every day or two,—the bath to be continued fifteen to thirty minutes, and the dressing to remain till it is replaced by another. He affirms that prompt cures were obtained by such treatment, in cases that had proved wholly intractable to ordinary remedies.

When, however, there was inflammation of the lining membrane of the neck (so graphically described by him, as we have seen), extending, as he surmised, in some cases, into the body itself, applications to its exterior merely were found inefficacious and hence he was induced to make trial of local remedies applied to the interior. To accomplish this, he made use of a hydrocele syringe with a gum elastic tube, which was introduced into the cervix a short distance above its external orifice, with a view of injecting its cavity, first with simple water to absterge it of its viscid mucus, and then with aqueous solutions of medicinal agents according to the indications to be fulfilled. He had reason to believe that the entire cavity of the womb was injected by his procedure. Sometimes there was reflux of the injected fluid before the tube was withdrawn; in other cases, it was retained until expelled by uterine contraction, accompanied with sharp pain of transient duration, followed by no accidents or dangerous consequences. M. MELIER reports favorably of these uterine injections though he states expressly that the cases in which they were employed were very protracted and difficult to subdue,—a confession which proves his good faith to the satisfaction of every one practically conversant with such cases.

Since the publication of M. MELIER'S memoir, injection of the uterine cavity has been resorted to by many practitioners, some of whom profess to have obtained satisfactory results from it, whilst others question its safety as well as its utility. In 1840, M. VIDAL (DE CASSIS), who is a great champion of the practice and has charge of a female hospital in Paris, the *Lourcine*, published an essay on the subject,* in which he endeavors to account for the different results and conflicting opinions, which he ascribes to—

1st. Difference of procedure.

2nd. Errors of diagnosis.

3rd. An erroneous interpretation of the symptoms consequent to these injections.

To obviate the objection that there is danger of the injection passing along the Fallopian tubes and thus penetrating into the peritoneal cavity, he performed a number of experiments on the dead body, characterised, according to the manner in which they were performed, as—

1st. Forcible injections.

2nd. Abundant injections.

3rd. Moderate injections.

It would not be interesting or instructive to transcribe the details of these experiments; their result may be briefly expressed. The subjects were women of different ages, most of whom had borne children. In some, the uterus and its appendages were left in their natural connections; in others, they were removed from the body. Both large and small syringes were used for the injections, with long canulas inserted in the mouth of the womb, secured, in some, by ligatures around the cervix to prevent reflux

* Essai sur un Traitement Méthodique de quelques Maladies de la Matrice.

of the fluid. *When a large syringe was used and the injection was made forcibly or abundantly, it often penetrated into the uterine veins and sometimes exuded by one or both Fallopiau tubes. When, however, the injection was made with a small syringe, and of course in moderate quantity and with little force, it always returned by the mouth of the womb by the side of the canula and never passed into the Fallopian tubes or reached the peritoneal cavity.*

These experiments indicate, as M. Vidal thinks, the kind of injections which ought to be made in the living, and accordingly he recommends for the purpose a *bivalve speculum*, *a small syringe*, not containing above twenty grammes of liquid, and a *small silver canula*, with several little holes in its bulbous extremity. Care is to be taken to expel the air from the syringe, and other precautions are also inculcated by him, such as,—

1st. Injections ought not to be practiced three days before the approach of the menses or three days after their cessation.

2nd. They ought to be deferred six months after accouchement or abortion.

3rd. They ought to be taken on an empty stomach (*La femme devra être à jeun*). M. Vidal, moreover, usually prepares the patient for these injections, which he distinguishes as *intra-uterine*, by injections thrown with great force from a large syringe on the os uteri, having previously exposed the part by the introduction of a bivalve speculum, and this is his *intra vaginal* injection. External affections of the cervix he treats by *intra-vaginal* injections, which may be repeated daily, and his favorite injection of this kind is a decoction of walnut leaves. The *intra-uterine* injections are not repeated so frequently, and a weak aqueous solution of iodine and the iodide of potassium, viz: a half grain of the former and one grain of the latter to the ounce of water, is most commonly preferred by him.

The *effects* produced by uterine injections are, according to M. Vidal, very variable. Some females experience no pain, either immediate or consecutive, whilst others complain, at the moment, of a burning sensation in the womb or pain in the iliac regions, which either gradually abates or increases in intensity. If no pain is felt at the time, the patient may be attacked with a violent abdominal pain or cholic, an hour after the operation, accompanied with so much tenderness and febrile reaction as to simulate peritonitis, for which it has been mistaken; but M. VIDAL insists that the phenomena are purely nervous and will subside, in a day or two, without the employment of antiphlogistic remedies, which indeed do not even abridge their duration. Dr. ASHWELL is of a different opinion. In his chapter on leucorrhea, he relates several cases of what he deemed hysteritis consequent to uterine injections of a mild kind; in one of them nothing but tepid water was used, which, however, was followed by such "marked evidence" of hysteritis, as to call for bleeding, both general and local, purgatives, fomentations, and a strict antiphlogistic regimen.*

In my own practice I have not resorted to uterine injections for the last several years, having been deterred from their employment by the violent and apparently alarming symptoms which were occasioned by them in a few of my cases. The symptoms were sudden severe pain in the uterine region, accompanied with cramps, coldness of the extremities, and depression of the pulse. Brandy and laudanum, repeated at short intervals, together with frictions and sinapisms to the extremities, afforded relief, in the course of a few hours, and no injurious consequences ensued. But I was reluctant to incur the risk of such sudden alarms and agitations, even for the sake of all the benefit that might be expected from the practice. This was the more to be regretted, as I had unequivocal evidence of the efficacy of the treatment where it could be borne without these alarming effects.

Considering the subject in all its bearings, it occurred to me that such sudden and violent symptoms must be owing more to the *mode* in which the remedies were applied than to actual intolerance of the internal surface of the uterus. Acting upon this view, instead of abandoning the use of topical remedies altogether, I began to introduce them upon strips of lint, pushed into the uterine cavity with a probe or sound. I first applied the nitrate of silver in this way, notwithstanding that experience had taught me that a weak solution of it,—two grains to the ounce of water,—

* A Practical Treatise on the Diseases peculiar to Women.

injected into the uterus, might be followed by the alarming symptoms that have been detailed. I used, in commencing, a very weak solution, carefully prepared by the apothecary, and finding that it caused no more pain than an ordinary cauterization of the os uteri, I was emboldened to make it stronger and stronger, until I ceased to have it prepared by weight and measure, but took a strip of lint, wet it thoroughly with water, and passed the stick of caustic over it till it was imbued with, as I judged, a saturated solution. I have cauterized the internal surface of the womb in this manner, in quite a considerable number of cases, without any of the alarming consequences incident to intra-uterine injection. No practitioner hesitates, in cervicitis, to push the nitrate crayon into the neck to cauterize the whole extent of its internal surface. Experience warrants me to declare that we may, with as little hesitation, treat the internal surface of the body in the same manner, only a saturated solution is preferable to the stick, on account of its liability to break and be retained in the cavity,—an accident which sometimes happens in the neck. All the local remedies that are had recourse to in affections of the neck may be applied in the same way to the body, not excepting even the *potassa cum calce*, as intimated in my first lecture, though, to avoid being even the remote cause of mischief, I must say again that so powerful an article needs great circumspection in its use; it should be applied only for a few seconds, lest it burn deeply and produce irreparable lesion of the organ. Either as an application to the *interior* of the neck or the body, it must be allowed to be a most unsafe remedy, in careless or unskillful hands. It may be objected that an article so potent and liable to abuse ought not to be used at all in the treatment of uterine disease. To this I reply that inflammation of the genital mucous membrane is sometimes so inveterate, especially when associated with enlargement and induration of the organ, that no milder means will make any remedial impression upon it. The effects of this agent in such cases, when it is, judiciously managed, are indeed admirable. Under its transforming influence, the inflammatory vice is eradicated, probably by the dissolution of the old membrane and the production of a new one, while the deeper seated tissues are restored to a healthy or at least to a healthier condition.

A word or two of explanation may be needed in regard to the manipulation I practice. The only instruments necessary are a bivalve or tubular speculum and a Simpson's uterine sound, slightly bent at the first knob, two and a half inches from its point. The speculum being properly introduced, take a strip of lint, four or five inches in length and about a quarter of an inch in breadth (narrower or wider according to the patulousness of the uterine canal), and after saturating it with the solution to be applied, take it by one of its extremities with the speculum forceps and lay it upon or a little within the os uteri. Then insinuate the sound into the uterine orifice, carrying the lint before it, and introduce it rapidly into the cavity, lest the contraction excited by the application should offer some impediment to its complete introduction. When the sound is inserted the whole length of the cavity (about two and a half inches) one extremity of the lint will be pendent at the uterine orifice, and by this it may be easily extracted with the forceps, should it chance to remain in the cavity aster the withdrawal of the sound. The lint may be held in the uterus a minute or two, when any article is used except the *potassa cum calce*, and its contact with every part of the cavity may be promoted by turning the sound a few times on its axis.

